# Partner's Education and Mortality in Finland: A Study of Married and Cohabiting Unions Among Cohorts Born Between 1932 and 1970

**DOI:** 10.1007/s10680-025-09752-8

**Published:** 2025-10-27

**Authors:** Cecilia Potente, Lydia Palumbo, Marika Jalovaara

**Affiliations:** 1 Erasmus School of Health Policy and Management, Erasmus University, Rotterdam, The Netherlands; 2 INVEST Flagship, University of Turku, Turku, Finland

**Keywords:** Educational assortative mating, Mortality inequalities, Resource multiplication mechanism, Cohabitation and marriage dynamics, Socioeconomic health disparities

## Abstract

The consequences of educational expansion and changes in couples’ educational distribution on mortality risk remain understudied. Using Finnish full population register data, this study examines the extent to which the education of both partners in married and cohabiting couples born between 1932 and 1970 is related to mortality risk. The results of Gompertz survival models show that a “resource multiplication mechanism” tends to prevail among these couples. Specifically, homogamous highly educated couples tend to have the highest survival advantage, low-educated couples have the greatest mortality risk, and heterogamous couples fall in between. One exception is women born in 1932–1950, who present a “resource substitution mechanism.” Women in couples in which one of the partners has a low level of education have similar survival probabilities to those in highly educated couples, meaning that men’s education could fully compensate for women’s lack of education. However, among women born between 1951 and 1970, these differences grow to resemble those observed in men, although they remain less pronounced. Furthermore, mortality has risen over time among low-educated couples, particularly cohabiters, while highly educated married couples have experienced significant mortality declines. Overall, cohabiters and low-educated men partnered with low-educated women emerge as the most vulnerable groups.

## Introduction

The expansion of women’s educational attainment and their increased participation in the labor market in the second half of the twentieth century marked significant socioeconomic changes in modern societies (Blossfeld, 2009; Van Bavel 2012). For instance, the proportion of women participating in tertiary education in North America and Europe increased from 39 to 56% since the 1970s, first matching and then surpassing that of men (De Hauw et al., 2017; Parvazian et al., 2017). Similarly, female labor force participation has increased in recent decades to encompass 60–70% of the female working-age population (Eurofond, 2016; Toossi & Morisi, 2017).

Several demographic studies highlight the strong connection between education, labor market positioning, and individual health (Ross & Mirowsky, 2006; Smith et al., 1998; Torssander & Erikson, 2010). However, it is reasonable to assume that individual socioeconomic circumstances also affect the health of those in close relationships. First, individuals’ health is deeply embedded within the context of their most intimate relationships, such as those with partners or spouses, meaning their partner’s health is likely to be influenced (Kilpi et al., 2018; Syse & Lyngstad, 2017; Torssander & Erikson, 2009). Second, an individual’s socioeconomic position is determined by not only their own resources, such as education, income, or occupation, but also by their married or cohabiting partner’s socioeconomic resources (Blossfeld & Drobnič, 2001; Blossfeld & Timm, 2003). In various Western contexts, studies have identified a correlation between one partner’s socioeconomic condition and the health of the other (Brown et al., 2014; Kilpi et al., 2018; Monden et al., 2003; Skalická & Kunst, 2008; Syse & Lyngstad, 2017; Torssander & Erikson, 2009; Torssander et al., 2018). Nevertheless, few studies have considered the role of one partner’s characteristics in influencing the other’s mortality (e.g., Skalická & Kunst, 2008; Torssander & Erikson, 2009).

Educational inequalities in all-cause mortality have widened over time (Long et al., 2023; Ma et al., 2012), particularly when viewed from a cohort perspective. For instance, in the US and Europe, socioeconomic disparities in mortality, especially deaths from heart disease and lung cancer, have steadily increased across cohorts (Long et al., 2023; Masters et al., 2012; Montez et al., 2019). This trend is also evident in Northern European countries, where established social democratic welfare systems are expected to mitigate such inequalities more effectively (Esping‐Andersen, 2002). For instance, Zarulli et al. (2012) identified a growing educational gap in life expectancy in Sweden and Finland, whereas Shkolnikov et al. (2012) documented both absolute and relative mortality inequalities in Finland, Norway, and Sweden.

Two theoretical mechanisms, originally developed at the individual level, resource substitution and resource multiplication, can be extended to analyze the relationship between mortality and couples’ educational attainment (Ross & Mirowsky, 2006; Stauder et al., 2019). The substitution mechanism posits that the health disadvantages associated with one partner’s low education can be offset by the higher education of the other partner. Conversely, resource multiplication theory suggests that partners’ education levels interact to amplify health outcomes. A highly educated partner enhances the relationship quality and health benefits through greater economic and social resources, while a less-educated partner offers fewer resources, thereby limiting potential health benefits (Stauder et al., 2019).

Heterogeneity in these mechanisms may arise depending on the type of coresidential relationships, such as marriage or cohabitation. These two relationship types have been shown to involve different socioeconomic resources (Lundberg et al., 2016), even in Nordic countries (e.g., Finland: Jalovaara & Andersson, 2018). With shifts in women’s socioeconomic status and the growing prevalence of cohabitation, the distribution of partners’ educational resources across coresidential relationships likely varies across cohorts.

Given the changes in socioeconomic, demographic, and health dynamics, this study investigates how partners’ educational levels are associated with individual all-cause mortality risk across birth cohorts in Finland. We also examine whether this relationship differs between married and cohabiting couples. Prior studies have  primarily focused on individual-level characteristics and rarely considered couple-level attributes. Furthermore, little attention has been paid to the intersection of mortality, socioeconomic disparities, and cohort and partnership type.

Using the Finnish full population register data from 1987 to 2020, we examined married and cohabiting individuals aged 50–88 years (the oldest available observation). Focusing on the observations of individuals aged 50 years or older allowed us to target a population subgroup with a higher mortality risk compared to children and younger adults. By leveraging population data, our research contributes to the understanding of the mechanisms underlying the socioeconomic and health inequalities of partnered individuals, who constitute a significant majority of the population (Statistics Finland, 2024b).

Finland serves as a valuable case study applicable to other contexts, as it aligns with trends that are observed in other high-income countries. First, despite universal healthcare coverage and a generous welfare system (Aspalter, 2020), Finland reflects similar socioeconomic mortality inequalities found in other low-mortality contexts (Mackenbach et al., 2018). Furthermore, Finland mirrors typical patterns of change in educational assortative mating in Europe (De Hauw et al., 2017) and is therefore a valuable case for studying the consequences of the expansion of higher education among men and women. However, Finland also exhibits unique characteristics typical of Nordic countries, which are leaders in gender equality. These forerunners of gender equality lead us to expect that women’s education may exert a greater influence on male mortality than in other high-income countries (Rijken & Liefbroer, 2016).

## Background

This study aligns with the key principles of the life course developmental theory (Elder, 1994; Elder et al., 2003). First, the “lifespan development” principle is consistent with the idea that health inequalities develop through the accumulation of advantages and disadvantages across different life stages (Kuh & Ben-Shlomo, 2004). Health outcomes are determined by various social, biological, and economic factors that create a long-term cumulative advantage or disadvantage (Halfon & Hochstein, 2002; Halfon et al., 2022). Another relevant principle is “linked lives”, which emphasizes the interdependence of individuals’ lives over the life span through relationships with family, friends, and co-workers. Consequently, an individual’s health should not be studied in isolation but rather in the context of their familial or social connections, including married or cohabiting partners (Holt-Lunstad, 2018). This underscores the importance of examining the role of partners’ socioeconomic status (SES) from both a life course and a couple perspective (Carr, 2018). Finally, the life course theory highlights the critical role of time, whether through the aging process or “historical time,” which reflects the period in which individuals are born and live. Thus, when examining health across the life course, it is essential to consider the interplay of age, period, cohort effects, and combinations of these factors (Bell, 2020).

### Partner’s Socioeconomic Status, Health, and Mortality

The literature identifies three main pathways through which a partner’s SES may impact the other partner’s health: material, psychosocial, and behavioral. First, a partner with higher SES provides better material resources for the entire household. For instance, higher SES is associated with greater employment opportunities (Bartley & Owen, 1996), and, among those who are employed, better employment quality, such as higher income and improved working conditions (Hoven & Siegrist, 2013). Second, partners with higher SES often possess greater psychosocial resources, including enhanced social and emotional support and a stronger sense of control over life (Monden et al., 2003). These resources can contribute to better health outcomes. Finally, higher partner SES is linked to healthier behaviors in couples, which may also improve overall well-being and health (Stauder et al., 2019). Examples include improved health literacy, engagement in preventive health screenings, and mutual monitoring of health-related habits (Jackson et al., 2015; Margolis & Wright, 2016; Monden et al., 2003).

Education is the most frequently used indicator of SES in health research, although income and employment status are also commonly studied (Torssander et al., 2018). Different socioeconomic indicators may affect a partner’s health through distinct mechanisms. For instance, a study in Norway analyzed the separate contributions of husbands’ and wives’ socioeconomic indicators and suggested that women’s education plays a role comparable to men’s occupational class and income (Skalická & Kunst, 2008). For example, one partner’s income can positively affect the other partner’s health by providing the financial resources necessary for effective management of a disease. Similarly, one partner’s education could benefit the other partner’s health by promoting literacy about healthy behaviors and encouraging behaviors that delay the onset of illnesses. Studies in Israel, Norway, and Sweden have examined the relationship between spousal education and mortality, consistently finding stronger effects in men than in women. This suggests that education significantly influences health behaviors (Jaffe et al., 2006; Kravdal, 2008). Additionally, previous studies on socioeconomic resources and health reveal that education is more strongly associated with the timing of disease onset, whereas income is closely related to disease progression (Herd et al., 2007; Zimmer & House, 2003).

#### H1

High levels of individual and partner education are associated with greater individual survival probability, than low levels of education.

### Assortative Mating, Health, and Mortality

Understanding the cross-spousal effects of SES is increasingly relevant in light of the evolving patterns of assortative mating. Educational expansion has provided new opportunities for young people to meet potential partners with similar characteristics (Blossfeld, 2009). Educational homogamy, defined as “the heightened tendency to mate with individuals with similar levels of education more frequently than would be expected under random circumstances” (Permanyer et al., 2019), has risen significantly over the twentieth century. This increase in homogamy is largely attributable to the increasing number of women pursuing post-secondary education (Hou & Myles, 2008) and the declining frequency of intermarriages between college-educated individuals and those without a high school diploma (Schwartz & Mare, 2005). Research indicates that, over time, individuals are selecting potential partners based less on ascribed characteristics, such as parental SES, and more on achieved ones, such as education and occupation (Blossfeld, 2009; Kalmijn, 1994; Mäenpää, 2015; Mäenpää & Jalovaara, 2015; Rosenfeld, 2008; Rosenfeld & Thomas, 2012; Wagner et al., 2020). Consequently, assortative mating across different partnership contexts has led to profound societal changes, warranting closer analysis regarding its implication for changes in mortality.

When analyzing partners' educational levels, it is essential to acknowledge that each partner contributes distinct types of resources. Two individual-level theoretical frameworks, resource substitution and resource multiplication, have been used to explore the interplay between different types of resources and health (Ross & Mirowsky, 2006). These frameworks have also subsequently been applied to explain how the distribution of education within couples impacts each partner’s health (Stauder et al., 2019). Resource substitution theory posits that one resource can effectively compensate for another without adverse effects on health outcomes. According to this framework, health and mortality outcomes are not dependent on any specific resource (Ross & Mirowsky, 2006; Stauder et al., 2019). For example, if education is viewed as a substitutable resource, one partner’s higher education could offset the other partner’s lower education. Thus, under the resource substitution framework, couples in which both partners are highly educated would have a similar survival probability to those in which only one partner is highly educated.

Resource multiplication theory suggests a direct and additive relationship between the number of resources and health outcomes (Ross & Mirowsky, 2006). According to this perspective, a partner's health benefits are maximized when both their own and their partner’s education levels are high. Highly educated individuals are more likely to partner with other highly educated individuals, compounding their health advantages. If resource multiplication is verified, couples in which both partners are highly educated would experience greater health benefits than those where only one partner is highly educated.

Despite these theoretical advances, the literature has largely overlooked how changes in assortative mating influence health inequalities at the individual level. Limited research exists on how both partners’ educational levels jointly impact each partner’s health. The effects of homogamy (both partners having similar education levels) versus heterogamy (partners with differing education levels) on all-cause mortality have been studied in only two contexts: Germany and the USA (Fan & Qian, 2019; Stauder et al., 2019). Fan and Quian (2019) employed simulation methods to assess how changes in educational homogamy within married couples in the US affected mortality inequalities over time. Their findings indicated that educational homogamy played a limited role in shaping mortality. Stauder et al. (2019) investigated the relationship between partner education, educational homogamy, and individual health among married individuals and cohabitants in Germany. Their study revealed that partner mortality was significantly lower in couples where both partners were highly educated. Additionally, they discovered that women’s mental and physical health benefited from having highly educated partners.

### Partners' Education, Coresidential Partnerships and Cohort Changes Over Time

Research consistently demonstrates that marital status is a critical determinant of health and mortality in high-income countries (Franke & Kulu, 2018a, 2018b; Josefsson et al., 2018; Kilpi et al., 2015; Koskinen et al., 2007; Kravdal et al., 2018; Martikainen, 1995; Silventoinen et al., 2022). Individuals in coresidential partnerships—whether cohabiting or married—tend to have longer survival rates compared to single individuals, whether previously partnered or never partnered (Kravdal, 2008). This survival advantage is often attributed to a combination of selection effects (healthier individuals are more likely to partner) and protective effects, such as mutual encouragement to avoid risky behaviors, shared resources, and emotional and social support (Kulu et al., 2024).

Despite these general benefits, the resources derived from coresidential partnerships vary significantly between cohabitation and marriage, with implications for health and mortality outcomes. Married couples often exhibit better health and lower mortality rates than cohabiting couples (Drefahl, 2012; Franke & Kulu, 2018a; Kravdal et al., 2023; Kulu et al., 2024). This disparity can be attributed to the longer-term commitments inherent in marriage, which require more substantial resource investment (Sassler & Lichter, 2020). Married households tend to have more resources, either through the selection of partners with higher SES or through the so-called marriage premium, whereby married men, in particular, enjoy improved economic and health outcomes (Kravdal et al., 2023). Furthermore, the higher average relationship quality in marriages may foster stronger mutual supervision and support (Kravdal et al., 2023). It is also important to highlight that pre-marital and post-marital cohabitation (re-partnering after separation or widowhood) in elderly ages differ in terms of socioeconomic resources. Lindmarker et al. (2025) show that, in Sweden, long-term cohabitation without marrying is more likely to represent a disadvantaged state than cohabiting after being married, and, therefore, more likely to be linked to higher mortality. The dynamics of assortative mating also differ between marriage and cohabitation. Studies in the USA have found that cohabiting couples are generally more homogamous than married couples, reflecting the “looser bond theory,” which concludes that cohabiters prioritize more egalitarian and similar partners (Laufer & Gemici, 2011; Schoen & Weinick, 1993). Conversely, other studies have found minimal or no differences between married and cohabiting couples, supporting the “informal marriage” perspective, particularly in contexts like the USA and the Netherlands, where cohabitation has become widespread (Blackwell & Lichter, 2000; Verbakel & Kalmijn, 2014). However, in countries with limited cohabitation diffusion, such differences persist (Hamplova, 2009).

In Finland, partnership formation underwent significant transformations earlier and more rapidly than in many other Western nations. Among cohorts born before the 1950s, direct marriage (without preceding cohabitation) was the predominant form of coresidential partnership (Lindgren et al., 1992). Cohabitation was largely limited to selective groups, such as divorcees, non-religious individuals, and those in non-normative relationships (Aromaa et al., 1983; Lindgren et al., 1992).

Simultaneously, despite Finland having the highest proportion of women attending tertiary education among other Nordic countries (Husu, 2007) and their full integration into the labor market, men’s socioeconomic resources remained more influential within marriages (Klement & Rudolph, 2004). This dynamic often meant that, in alignment with previous literature (e.g., see Becker, 1993), marriage predominantly entailed that men were more highly educated than women and served as primary income contributors, shaping health outcomes differently for men and women within marriages (Aromaa et al., 1983). Thus, married women's health outcomes were more likely to be influenced by men's SES than their own. In contrast, men's health outcomes were more likely to be affected by their own resources than those of women. Cohabitation in Finland diverged from this traditional marital model (Aromaa et al., 1983). Both partners in cohabiting relationships were more likely to benefit from their own resources and their partners’ socioeconomic resources, regardless of sex.

Starting in the 1960s, Finland pioneered a marked decline in marriage rates alongside a corresponding rise in cohabitation, trends mirrored across the Nordic region (Finnäs, 1995). Unmarried cohabitation overtook direct marriage as the dominant way of entering the first coresidential partnership. Only one-third of women born in the 1960s were married by the age of 25, whereas they were two-thirds among women born in the early 1950s (Finnäs, 1995). In recent decades, cohabitation also developed characteristics akin to marriage, such as longer durations and the increased prevalence of children born to cohabiting couples (Mäenpää & Jalovaara, 2015; Rahnu & Jalovaara, 2023). However, cohabitation remains less stable than marriage, with most unions (approximately 75%) among Finns aged 18–35 ending in either marriage or separation within five years from the start of cohabitation (Jalovaara, 2013).

Socioeconomic differentiation, in Finland, is more pronounced in entry into marriage than in the one for cohabitation. While cohabitation tends to be less stratified by education and more common among younger cohorts as a precursor to marriage (Jalovaara & Fasang, 2015), highly educated individuals are more likely to enter marriage by age 39 and less likely to experience non-marital childbearing (Jalovaara & Andersson, 2018, 2023; Jalovaara & Fasang, 2015; Schnor & Jalovaara, 2020). These disparities reflect the higher demands associated with marriage compared to cohabitation. Among recent cohorts, cohabitation has become a more egalitarian household model (Brines & Joyner, 1999), reliant on both partners’ resources, while marriage has also evolved toward greater gender equality in resource contributions (Jalovaara, 2012, 2013; Jalovaara & Miettinen, 2013).

For the most recent Finnish cohorts, both men’s and women’s socioeconomic resources have played a significant role in decisions to enter either cohabitation or marriage (Jalovaara, 2012, 2013; Jalovaara & Miettinen, 2013). Several international studies have shown that women’s marital resources have become more important over time (Blossfeld, 2009; Sweeney, 2002; Sweeney & Cancian, 2004). This suggests that the socioeconomic characteristics of both spouses are important to their outcomes. The reasons for this discrepancy may vary. Cohabiters transitioning to marriage, which were the majority, may have maintained more liberal and egalitarian values when they were married than individuals transitioning to marriage directly (Jalovaara, 2013). Furthermore, there could also be a compositional effect, as the share of men and women born in the 1960s with upper and post-secondary education increased, whereas those with lower education decreased. Women had much higher rates of post-secondary education than men, whereas men being secondary- or lower-educated were more than women across almost all cohorts born between the 1960s and 1980s (Härkönen & Sirniö, 2020). Given this, we hypothesize the following:

#### H2a

A resource substitution mechanism exists among married individuals born before or in 1950, differing by gender. Men’s higher education is associated with lower mortality risk for female partners, whereas women’s education does not significantly relate to male mortality risk.

#### H2b

Among married individuals born after 1950, a resource multiplication mechanism applies, meaning that each married partner’s mortality risk depends on both their own and their partner’s education.

#### H3

Within cohabitation, a resource multiplication mechanism holds for both genders and across birth cohorts.

## Data and Methods

This study utilized Finnish full population register data spanning 1987 to 2020. The analysis included individuals aged 50 years or older, who were thus exposed to the risk of dying from the beginning of 1988 onward. We focused on married and cohabiting individuals born in Finland between 1932 and 1970. The earliest birth cohort (1932) corresponded to those who were age 18 at the time Finland’s first Population Census was conducted in 1950 (Statistics Finland, 2024a). By contrast, the upper cohort boundary coincided with individuals turning 50 in 2020, the final year of observation in this study. Each individual meeting these criteria served as an anchor, linking their current co-resident partner and the partner’s characteristics. Anchor individuals (here, referred to as simply individuals) were permitted to have more than one partnership and, thus, more than one partner. We restricted the analysis to include only different-sex couples, as we were interested in the sex-specific effects of education on mortality in both partners.

The partners could have formed coresidential relationships either before or after 1987. While marriage statistics have been made available by Statistics Finland since 1950, cohabitation could only be inferred from register data starting at the date of the first census, namely from 1987. Finnish registers provide detailed information regarding the specific dwelling unit residents, making it possible to infer cohabiting couples, even if they are childless or unmarried (for more information regarding the inference of cohabitation, see the Supplementary Material by Jalovaara & Kulu, 2018). For this reason, the analyses were stratified by coresidential partnership (marriage or cohabitation) and sex.

Our final analysis relied on 926,060 men and 911,398 women. The number of deaths in our sample amounted to 201,464 (10.6% of the total number of couples). However, coupled individuals could exit for reasons other than the event of death. First, they may not have experienced death by 2020 (64.1% of the couples in the sample). Second, they may have become widowed (10.3% of couples) or divorced or separated during the observation period (14.1% of couples). Since these marital statuses were not included in our study, these observations were excluded from the data. Third, they may have emigrated (around 1% of the couples’ exits remain unclassified). The observations from individuals who separated, and later reentered partnerships were retained in the sample. Therefore, individuals with multiple partnerships were included in the analysis. We also included left-censored individuals, whose partnerships (either cohabitation or marriage) started before the beginning of the observation period.

We applied survival analysis to explore whether the individuals’ education and their partners' education are related to the risk of death among the individuals. We used a Gompertz survival model, which is a parametric model assuming hazard proportionality. The Gompertz model is named for its assumption that the baseline hazard, whose analysis time is here represented by individuals’ age (years), follows a Gompertz distribution with a shape parameter $\gamma$ (Cleves et al., 2010; Kalbfleisch & Prentice, 2002). The specification of the baseline hazard is the following:

$${h}_{0}\left(age\right)={e}^{\gamma age}{\text{e}}^{({\beta }_{0})}$$

Once entered in the proportional hazard model, the specification model is the following.

$$h(age|{x}_{j})={{h}_{0}\left(age\right)e}^{{x}_{j}{\beta }_{x}}={e}^{\gamma age}{\text{e}}^{({\beta }_{0}+{{x}_{j}\beta }_{x})}$$

The Gompertz survival model is commonly used in mortality studies for older adults. Its hazard rate increases monotonically in an exponential way over time (if $\gamma >0)$, as it occurs for human mortality (Cleves et al., 2010).

The base model of our study examined how, among married or cohabiting Finns aged 50 + , the individual’s mortality risk varied by own level of education, the level of education of the partner, and birth cohort. For this model, the Gompertz specification would be the following:

$$h(age|{x}_{j})={e}^{\gamma age}{\text{e}}^{({\beta }_{0}+{\beta }_{1}Edu:Int+{\beta }_{2}Edu:High+{\beta }_{3}Pa\_Edu:Int+{\beta }_{4}Pa\_Edu:High+{\beta }_{5}Birthc:1951-1970)}$$

where ${e}^{Edu}$, ${e}^{Pa\_edu},$ ${e}^{Birthc}$ refer to the hazard ratios of own education, partner’s education and birth cohort, respectively. The reference categories for these variables are, respectively,${e}^{Edu:Low}$, ${e}^{Pa\_edu:Low} , {e}^{Birthc:1932-1950}$.

Further, we analyzed a second model interacting the individual’s education with partner’s education. These models allowed testing our hypothesis H1. To test the subsequent hypotheses, we employed a model that included a three-way interaction among the individual's educational level, birth cohort, and the partner's education. Wald tests confirmed the appropriateness of introducing such interactions.

We computed separate models by the individual’s sex and the couple’s coresidential partnership (marriage or cohabitation). Because most individuals have more than one observation, due to the annual data registration, the standard errors were clustered at the individual level. Using this model, we calculated the survival curves for two cohort groups: an earlier cohort (1932–1950) and a later cohort (1951–1970). For the earlier cohort, results were graphed from ages 50 to 88, while results for the later cohort were graphed until age 65.

Given the assumption of hazard proportionality of the Gompertz model, we tested this assumption on our final model (three-way interaction), based on the rejected weighted scaled residuals of a Cox regression (Grambsch & Therneau, 1994). From the tests, we concluded there was not enough evidence to reject the proportionality assumption in three out of four models (the exception concerns married men). For this reason, we decided to employ a Gompertz model. Results of the tests are reported in Table 5 in the Appendix.

In our study, education was treated as a time-invariant variable, defined as the highest level of educational achievement by the age of 34. Education was categorized into three ordinal categories: (1) Low, including individuals with only compulsory education (ISCED 0–2); (2) Intermediate, encompassing secondary-level education, such as occupational training or the final examination at the general upper secondary school graduation (ISCED 3–4); and (3) High, covering tertiary education, which includes both lower tertiary (2–3 years post-secondary education) and degree-level tertiary education (ranging from bachelor’s to doctoral degrees from university) (ISCED 5–8) (Jalovaara & Miettinen, 2013; Jalovaara et al., 2022).

Birth cohorts were categorized into two groups: 1932–1950 and 1951–1970. These groupings reflect cohorts exposed to similar changes in partnership dynamics and socioeconomic contexts. As highlighted earlier, analyses were stratified by sex and partnership type. Partnership type was a time-varying variable that indicated whether individuals were married or cohabiting each year of their study inclusion.

## Results

Tables 1 and 2 summarize the distribution of time at risk, measured in person-years, by birth cohort and sex. In the 1932–1950 cohort, individuals are predominantly married (over 90% of the sample) rather than cohabiting. However, in the 1951–1970 cohort, the proportion of married individuals drops to 82.6–83.8%, which is expected given the larger diffusion of cohabitation in the second half of the twentieth century. By contrast, the proportion of cohabiting individuals has increased over time (from around 8% to 16.2–17.4%). However, the percentage of married person-years is still much larger than that of cohabiting person-years in both cohorts. Indeed, as many cohabiters still tend to transition to marriage before reaching older age, long-term cohabitation is rare (Jalovaara & Kulu, 2018).

**Table 1:** Descriptive statistics of individuals in partnerships in the sample, birth cohort 1932–1950

	Women				Men			
	Person-years	%	Deaths	%	Person-years	%	Deaths	%
*Partnership type*								
Cohabiting	716,539	8.5	3619	5.2	855,204	8.4	8188	9.9
Married	7,694,577	91.5	44,565	12.2	9,355,651	91.6	115,621	27.9
*Education*								
Low	7,053,491	83.9	42,475	14.3	8,259,440	81.0	108,675	32.6
Intermediate	529,999	6.3	2413	10.2	507,403	5.0	4618	21.2
High	827,626	9.8	3296	8.9	1,424,012	14.0	10,516	17.2
*Partner’s education*								
Low	6,791,110	80.7	40,886	11.8	7,798,644	76.5	103,573	27.2
Intermediate	463,777	5.5	2413	8.4	1,127,720	11.1	10,568	16.4
High	1,156,229	13.8	4885	8.2	1,264,491	12.4	9668	14.5

*Source*: Own computations from Statistics Finland register data (1987–2020)

(a) The same individual can be a partner in more than one couple. Individuals with more than one partnership were counted twice. (b) The percentage of deaths was computed on individuals, not person-years

**Table 2:** Descriptive statistics of individuals in partnerships in the sample, birth cohort 1951–1970

	Women				Men			
	Person-years	%	Deaths	%	Person-years	%	Death	%
*Partnership type*								
Cohabiting	790,872	16.2	2,010	1.7	813,295	17.4	4,409	3.9
Married	4,103,245	83.8	9,417	2.3	3,858,726	82.6	14,635	3.8
*Education*								
Low	1,137,412	23.2	3,975	3.9	1,181,848	25.3	6,516	6.0
Intermediate	1,984,767	40.6	4,546	2.1	1,948,094	41.7	7,457	3.6
High	1,771,938	36.2	2,906	1.4	1,542,079	33.0	4,071	2.6
*Partner’s education*								
Low	1,651,478	33.7	5,076	3.4	981,011	21.0	5712	6.4
Intermediate	1,722,103	35.2	3,729	1.8	1,898,014	40.6	7529	3.9
High	1,520,536	31.1	2,622	1.6	1,792,996	38.4	4803	2.5

*Source*: Own computations from Statistics Finland register data (1987 − 2020)

(a) The same individual can be a partner in more than one couple. Individuals with more than one partnership were counted twice. (b) The percentage of deaths was computed on individuals, not person-years

Regarding education, Table 1 shows that, in the earliest cohort, low-educated individuals and their partners account for the majority of person-years (81–84%). While few sex differences are noted for the low- and intermediate-educated groups, highly educated men tend to present more observations than highly educated women (14.0% vs. 9.8%). In the latest cohort, the proportion of low-educated person-years decreases markedly (by around 50–60 percentage points), accompanied by an increase in intermediate-educated and highly educated ones. For both sexes and cohorts, the percentage of deaths is the highest among low-educated partners and the lowest among the highly educated.

**Fig. 1:**
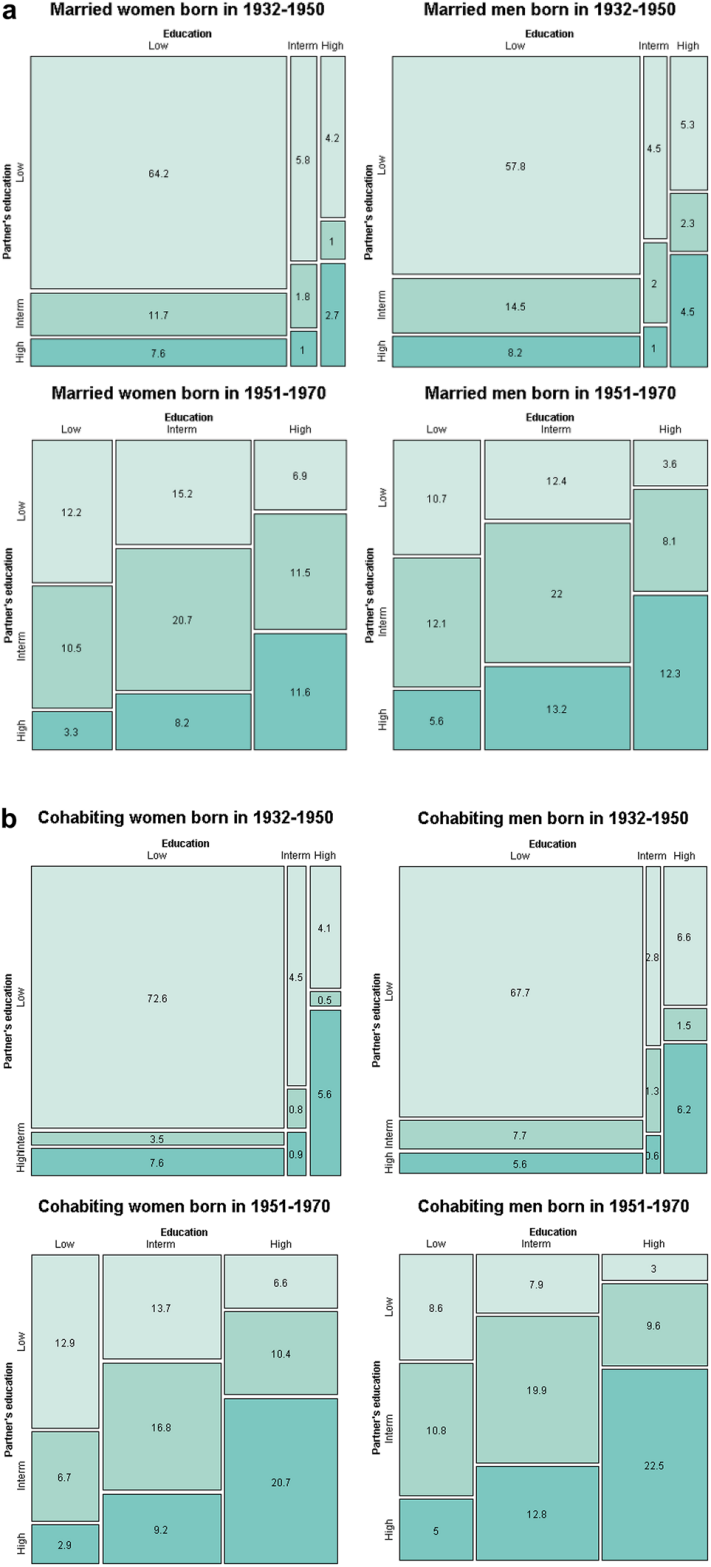
Percentage of unique ^a)^ marriages/cohabitation by women’s and men’s education and birth cohort*.* *Source*: Own computations from Finnish full population registers. *Note*: **a** By “unique marriages/cohabitations” we intend that we are counting each married or cohabiting couple once, regardless of how many times it appears in the dataset

Figure 1 presents mosaic plots of couples’ educational combinations by cohort, illustrating dramatic changes over time. Figure 1 shows that, in the 1932–1950 cohort, the majority of couples consisted of both partners being low-educated, particularly among cohabiting couples compared to married ones (e.g., among women, 72.6% within cohabitation and 64.2% within marriage). Marriage had a consistent share of intermediate-educated individuals with a low-educated partner (11.7% for women and 14.5% for men). Other pairings were rarer among cohabitants and married couples (less than 10%), with cohabitation presenting a slightly higher share of couples in which both partners were highly educated.

The subsequent cohort (1951–1970) was subject to changes due to educational expansion in the 1970s, when the share of low-educated individuals quickly declined, and that of secondary- and tertiary-educated individuals strongly increased (Statistics Finland, 2023). Figure 1 shows that the number of educational combinations in which one partner was either intermediate or highly educated has increased. In the case of marriage, the largest share of couples was represented by intermediately educated couples. Regarding cohabitation, highly educated individuals represented the highest proportion (20.7% of women and 22.5% of men).

Tables 3 and 4 provide the estimated hazard ratios for mortality risk of married and cohabiting individuals, respectively. First, to test H1, which hypothesizes that “High levels of individual and partner education are associated with greater individual survival probability than low levels of education”, we start with analyzing the results of Model 1. This model contains only the main effects of the individual’s education and that of their partner, while accounting for the individual’s birth cohort.

**Table 3:** Gompertz models for the mortality predictors of Finnish married individuals aged 50 + (hazard ratios)

	Men			Women		
	Model 1	Model 2	Model 3	Model 1	Model 2	Model 3
*Individual's education (ref. Low)*						
Intermediate	0.91***	0.95***	0.93***	0.90***	0.94***	0.96
	(0.89–0.93)	(0.92–0.98)	(0.90–0.97)	(0.87–0.93)	(0.90–0.98)	(0.92–1.01)
High	0.70***	0.69***	0.69***	0.76***	0.76***	0.77***
	(0.68–0.71)	(0.67–0.71)	(0.67–0.71)	(0.74–0.78)	(0.73–0.80)	(0.73–0.82)
*Partner's education (ref. Low)*						
Intermediate	0.91***	0.92***	0.91***	0.98	1.05**	1.08***
	(0.89–0.92)	(0.90–0.94)	(0.89–0.94)	(0.94–1.01)	(1.00–1.09)	(1.03–1.14)
High	0.74***	0.73***	0.73***	0.82***	0.81***	0.81***
	(0.72–0.75)	(0.71–0.75)	(0.71–0.75)	(0.79–0.84)	(0.78–0.84)	(0.77–0.84)
*Individual's education *$\times$* Partner's education*						
Intermediate $\times$ Intermediate		0.93***	1.04		0.85***	0.91
		(0.88–0.97)	(0.97–1.13)		(0.79–0.91)	(0.79–1.05)
Intermediate $\times$ High		0.91***	1.01		0.91**	0.94
		(0.86–0.97)	(0.91–1.13)		(0.84–0.99)	(0.81–1.08)
High $\times$ Intermediate		0.96	1.02		0.85***	0.92
		(0.91–1.02)	(0.95–1.10)		(0.77–0.93)	(0.76–1.11)
High $\times$ High		1.04	1.13***		1.03	1.15***
		(0.99–1.09)	(1.08–1.19)		(0.97–1.11)	(1.06–1.25)
*Birth cohort (ref. 1932–1950)*						
1951–1970	0.88***	0.89***	1.03	1.07***	1.07***	1.24***
	(0.87–0.90)	(0.87–0.91)	(0.99–1.07)	(1.04–1.10)	(1.04–1.11)	(1.18–1.30)
*Individual's education *$\times$* Birth cohort*						
Intermediate $\times$ 1951–1970			0.91**			0.84***
			(0.85–0.98)			(0.77–0.91)
High $\times$ 1951–1970			0.94			0.85***
			(0.85–1.04)			(0.76–0.94)
*Partner's education *$\times$* Birth cohort*						
Intermediate $\times$ 1951–1970			0.91***			0.81***
			(0.85–0.97)			(0.74–0.89)
High $\times$ 1951–1970			0.87***			1.00
			(0.79–0.95)			(0.89–1.13)
*Individual's education *$\times$* Partner's education *$\times$* Birth cohort*						
Intermediate $\times$ Intermediate $\times$ 1951–1970			0.93			1.12
Fi			(0.83–1.04)			(0.94–1.33)
Intermediate $\times$ High $\times$ 1951–1970			0.99			0.99
			(0.85–1.15)			(0.81–1.21)
High $\times$ Intermediate $\times$ 1951–1970			0.93			1.09
			(0.81–1.07)			(0.87–1.38)
High $\times$ High $\times$ 1951–1970			0.85**			0.81**
			(0.74–0.97)			(0.69–0.96)
Constant (${\beta }_{0})$	0.00***	0.00***	0.00***	0.00***	0.00***	0.00***
	(0.00–0.00)	(0.00–0.00)	(0.00–0.00)	(0.00–0.00)	(0.00–0.00)	(0.00–0.00)
Gamma parameter ($\gamma )$	1.10***	1.10***	1.10***	1.11***	1.11***	1.11***
	(1.10–1.10)	(1.10–1.10)	(1.10–1.10)	(1.11–1.11)	(1.11–1.11)	(1.11–1.11)
Observations	13,194,377	13,194,377	13,194,377	11,797,822	11,797,822	11,797,822

**Table 4:** Gompertz models for the mortality predictors of Finnish cohabiting individuals aged 50 + (hazard ratios)

	Men			Women		
	Model 1	Model 2	Model 3	Model 1	Model 2	Model 3
*Individual's education (ref. Low)*						
Intermediate	0.94**	1.07	1.12**	0.89***	1.04	1.20**
	(0.88–1.00)	(1.00–1.15)	(1.00–1.25)	(0.82–0.97)	(0.94–1.15)	(1.04–1.40)
High	0.69***	0.74***	0.77***	0.62***	0.71***	0.83
	(0.65–0.74)	(0.67–0.82)	(0.68–0.87)	(0.56–0.68)	(0.61–0.82)	(0.69–1.01)
*Partner's education (ref. Low)*						
Intermediate	0.84***	0.91***	0.94	1.00	1.17***	1.21***
	(0.80–0.89)	(0.85–0.96)	(0.88–1.01)	(0.93–1.07)	(1.07–1.27)	(1.09–1.35)
High	0.68***	0.74***	0.80***	0.73***	0.76***	0.82***
	(0.63–0.72)	(0.68–0.80)	(0.73–0.87)	(0.66–0.80)	(0.67–0.87)	(0.71–0.95)
*Individual's education *$\times$* Partner's education*						
Intermediate $\times$ Intermediate		0.75***	0.89		0.66***	0.88
		(0.67–0.84)	(0.71–1.11)		(0.57–0.77)	(0.63–1.22)
Intermediate $\times$ High		0.77***	0.97		0.8	0.66
		(0.66–0.89)	(0.72–1.32)		(0.63–1.00)	(0.38–1.15)
High $\times$ Intermediate		0.89	0.97		0.66***	0.85
		(0.76–1.05)	(0.76–1.24)		(0.52–0.83)	(0.53–1.37)
High $\times$ High		0.83**	0.75**		0.88	1.04
		(0.70–0.97)	(0.60–0.94)		(0.69–1.12)	(0.73–1.50)
*Birth cohort (ref. 1932–1950)*						
1951–1970	1.14***	1.14***	1.40***	1.66***	1.65***	2.10***
	(1.08–1.20)	(1.08–1.21)	(1.29–1.52)	(1.53–1.80)	(1.53–1.79)	(1.89–2.33)
*Individual's education *$\times$*Birth cohort*						
Intermediate $\times$ 1951–1970			0.79***			0.67***
			(0.68–0.92)			(0.55–0.82)
High $\times$ 1951–1970			0.77**			0.62***
			(0.61–0.96)			(0.46–0.82)
*Partner's education *$\times$* Birth cohort*			0.77***			0.78***
Intermediate $\times$ 1951–1970			(0.67–0.88)			(0.65–0.93)
			0.65***			0.67**
High $\times$ 1951–1970			(0.54–0.79)			(0.49–0.92)
*Individual's education *$\times$* Partner's education *$\times$* Birth cohort*						
			1.03			0.93
Intermediate $\times$ Intermediate $\times$ 1951–1970			(0.78–1.35)			(0.63–1.36)
			1.08			1.74
Intermediate $\times$ High $\times$ 1951–1970			(0.74–1.57)			(0.91–3.31)
			1.12			1.01
High $\times$ Intermediate $\times$ 1951–1970			(0.79–1.60)			(0.57–1.77)
			1.62***			1.12
High $\times$ High $\times$ 1951–1970			(1.13–2.32)			(0.66–1.90)
Constant	0.00***	0.00***	0.00***	0.00***	0.00***	0.00***
	(0.00–0.00)	(0.00–0.00)	(0.00–0.00)	(0.00–0.00)	(0.00–0.00)	(0.00–0.00)
Gamma parameter	1.10***	1.10***	1.10***	1.12***	1.12***	1.12***
	(1.10–1.10)	(1.10–1.10)	(1.10–1.10)	(1.12–1.12)	(1.12–1.12)	(1.12–1.12)
Observations	1,668,499	1,668,499	1,668,499	1,507,411	1,507,411	1,507,411

*Source*: Own computations from Statistics Finland register data (1987–2020)

(1) Confidence intervals in parentheses are set at the 95% level. (2) *P*-values signaled as follows: *** *p* < 0.01, ** *p* < 0.05; (3) Model 1 contains main effects only; Model 2 contains interaction between the individual's education and partner's education; Model 3 contains interaction between the individual's education, partner's education and birth cohort. (4) Constant terms are equal to zero due to rounding to two decimal digits

According to Table 3, a higher level of education is associated with a reduced mortality risk for married individuals. For instance, having an intermediate or a high level of education, rather than being low educated, is related to a significant decrease in mortality risk. Specifically, there is a 10% and 24% lower mortality risk for intermediate-educated and highly educated women compared with 9% and 30% reductions for men (Model 1). Therefore, the mortality risk reduction from having a high education is considerably larger than that from having an intermediate education.

Similarly, the higher the partner’s educational level, the lower the individual’s mortality risk. For married men, being partnered with an intermediate-educated woman is associated with a 9% lower mortality risk compared with being partnered with a low-educated woman. However, this mortality risk reduction is much larger if they are partnered with a highly educated woman (26%). Although we observe similar patterns for women, their mortality risk presents a weaker association with their male partners’ education than that of men. For instance, women have a 2% reduction in mortality risk if they have a partner with an intermediate education (not statistically significant) rather than a low-educated one and an 18% lower risk if they have a highly educated partner (significant at 1%).

The results of the cohabitation analysis reveal a similar pattern, with similar sized hazard ratios. As shown in Tables 3 and 4, men who partner with highly educated women have a 32% lower mortality risk than men whose partner has a low level of education. By contrast, the mortality risk of women who partner with highly educated men is 27% lower.

Using Model 2, we can verify the multiplicative effect of the individuals’ education and that of their partner on their mortality risk and, therefore, corroborate the validity of the first hypothesis. The results in Tables 3 and 4 suggest a multiplicative effect of the partner’s education on mortality risk for both men and women. In other words, there is a significant interaction between the individual’s education and that of their partner (significance confirmed by Wald tests). For instance, the hazard ratio of an intermediate-educated man born in 1932–1950 and married to an intermediate educated partner is 0.81 ($0.95\times 0.92\times 0.93)$, as shown in Table 3. By contrast, from the additive model (Model 1), the hazard ratio would be is 0.83 $(0.91\times 0.91)$.

**Fig. 2:**
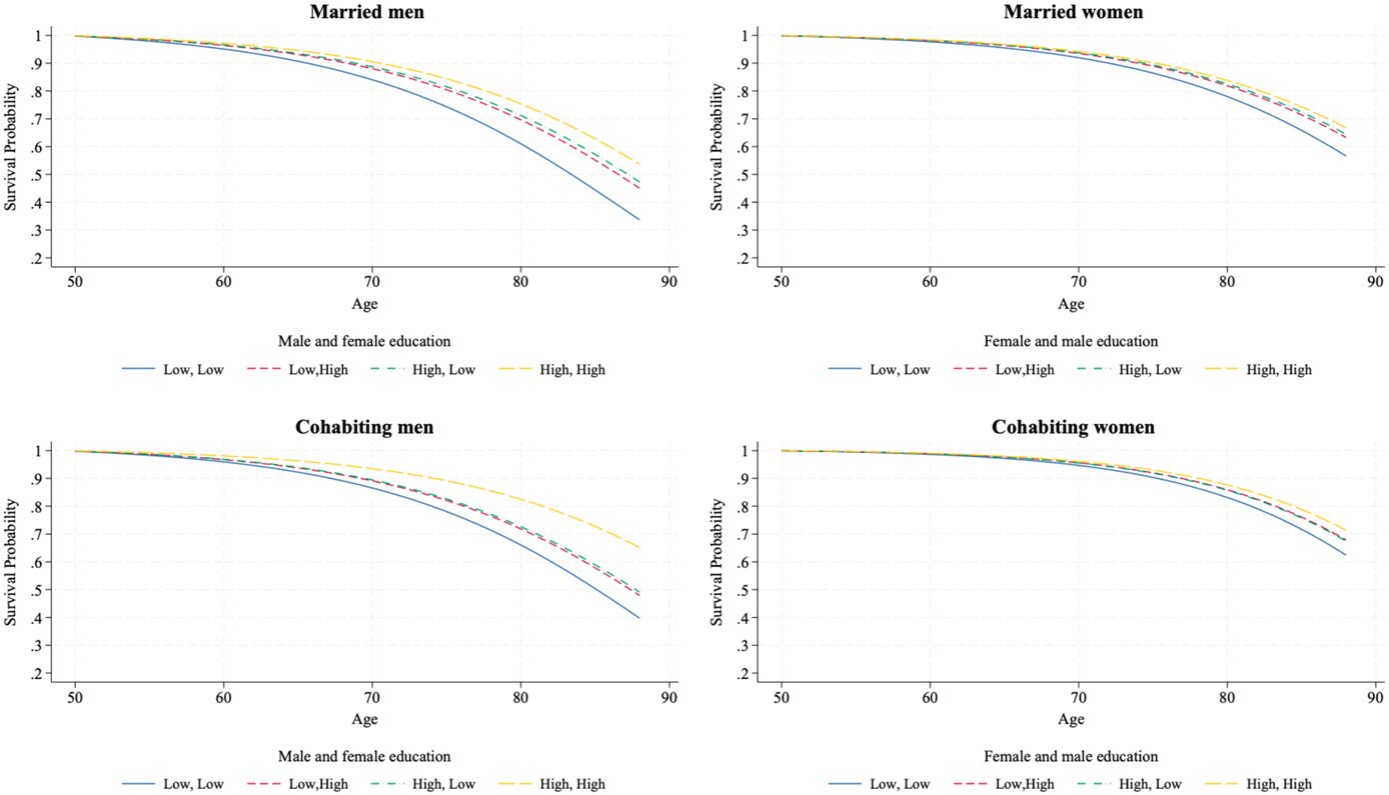
Survival curves for married and cohabiting individuals born in 1932–1950, by individual own education and partner’s education (from Gompertz models). *Source*: Own computations from Statistics Finland register data (1987–2020)

**Fig. 3:**
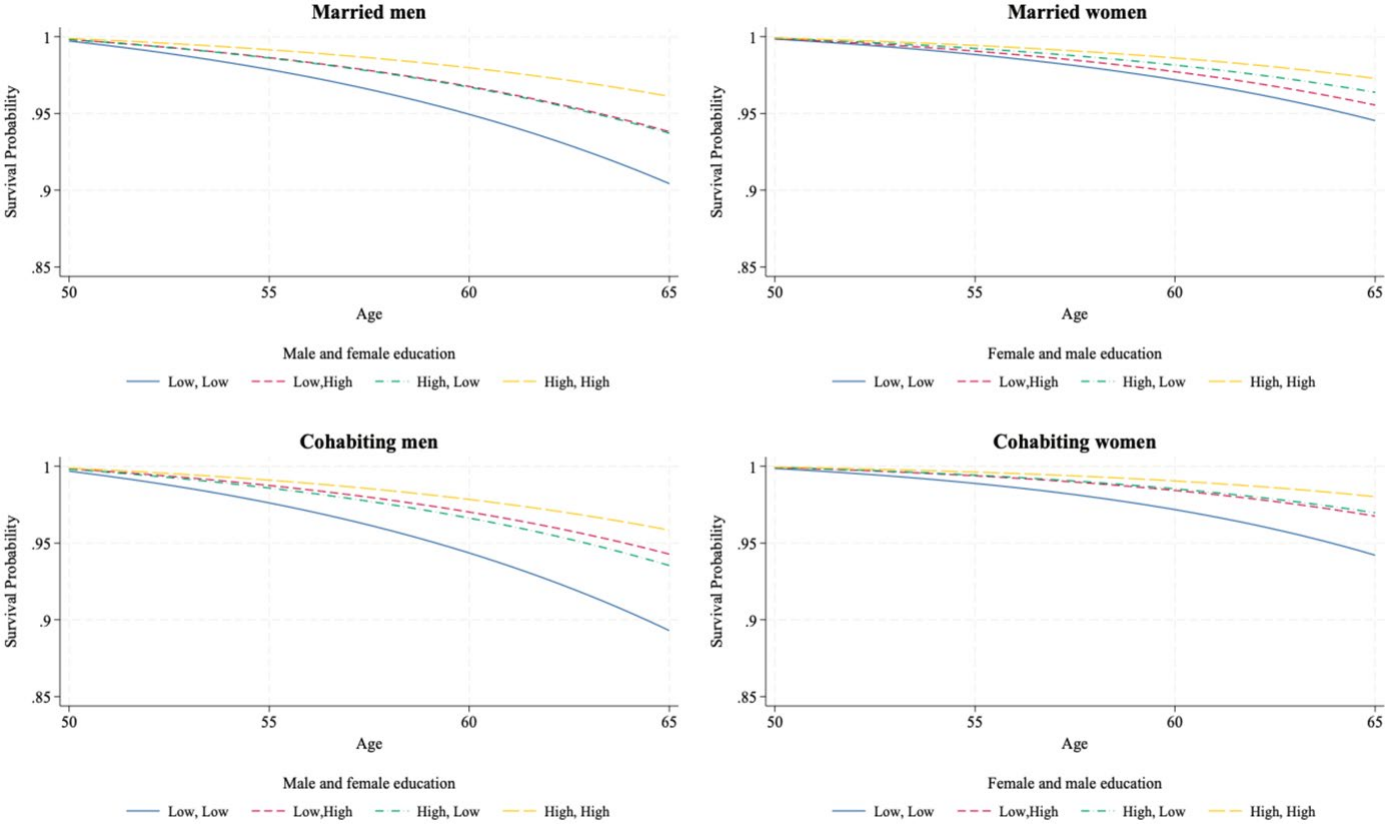
Survival curves for married and cohabiting individuals born in 1951–1970, by individual own education and partner’s education (from Gompertz model). *Source*: Own computations from Statistics Finland register data (1987–2020)

We now test H2a, H2b, and H3, which hypothesize that the effect of education on individuals’ mortality risk depends on their sex, their educational level and that of their partner, the birth cohort, and the coresidential union. We test these hypotheses using Model 3 in Tables 3 and 4, which includes the interaction term among the individual’s education, the education of their partner, and the birth cohort (the interaction is significant according to Wald tests), stratified by union and sex. To help interpret the new models, Figs. 2 and 3 illustrate the survival probabilities over age using survival curves for the different combinations of the individual’s education and that of their partner for the two birth cohorts. To improve readability, these graphs show only the survival curves of low-educated and highly educated individuals, as they show the largest differences in education. We then analyze the median survival time of different couple pairings to test the differences in survival across educational levels.

As shown in Fig. 2, among couples born in 1932–1950, the survival probabilities are significantly different between the lowest- and highest-educated couples, particularly among men. By age 88, highly educated men married or cohabiting with highly educated women are predicted to have a survival probability 20 to 25 percentage points higher than that of couples in which both partners have a low level of education. For married and cohabiting women, this difference in survival probability at age 88 is approximately 10 percentage points, from around 70% to 60%. Figure 2 also shows that heterogamous couples have a survival probability between the lowest- and highest-educated couples. These differences are far larger for men, as the curves of women in heterogamous couples are extremely close to each other and to those for which both partners are highly educated.

Figure 3 illustrates the findings for the 1951–1970 cohort until age 65, which is the age reached by around half of the individuals in this subsample. This cohort shows more pronounced differences in survival probability between highly educated and low-educated couples, particularly among women, where survival probabilities are more differentiated than in the earlier cohort.

**Fig. 4:**
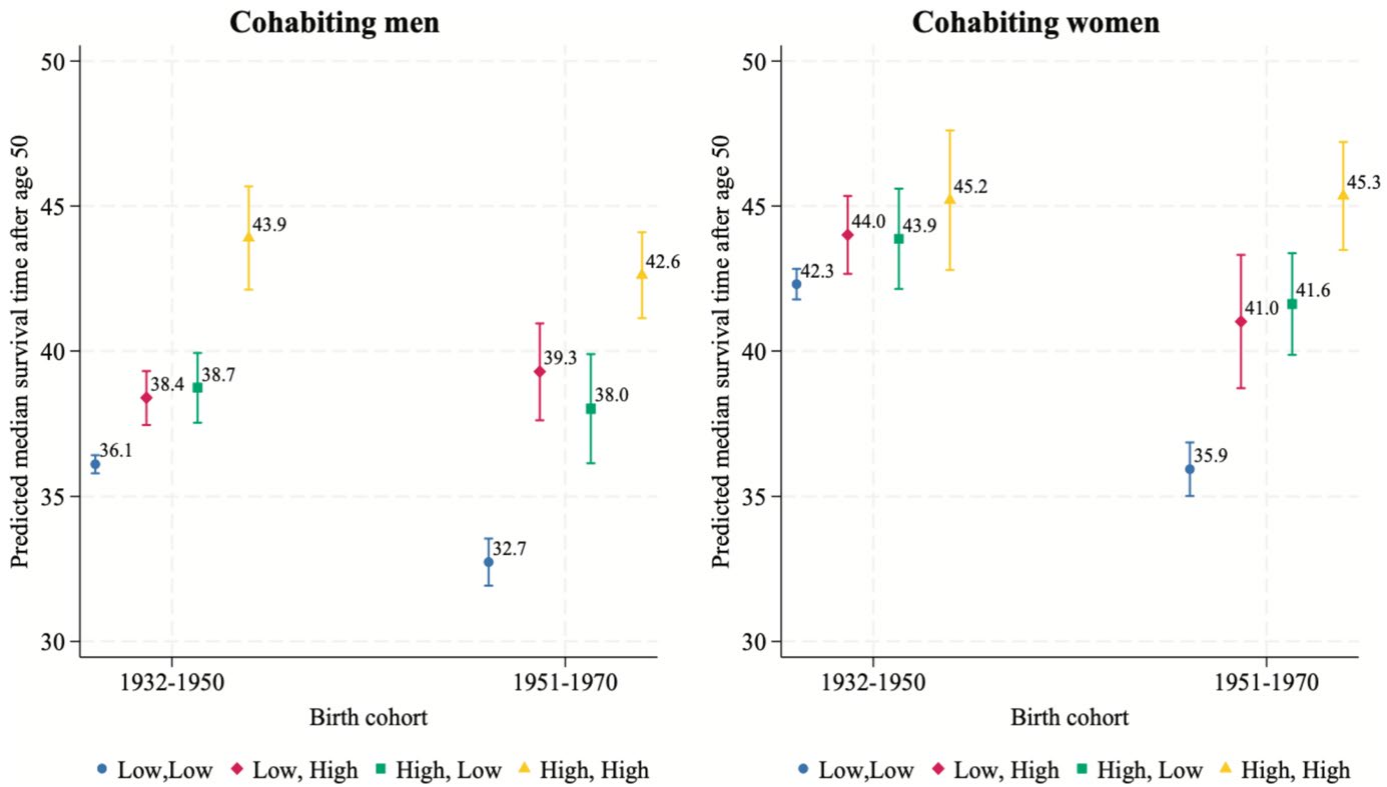
Predicted median time of survival for married individuals by educational pairing, gender and cohort. *Source*: Own computations from Statistics Finland register data (1987–2020). *Note*: (1) Confidence intervals are at the 95% level; (2) Numbers next to bars refer to median prediction. CIs available in Table 6

**Fig. 5:**
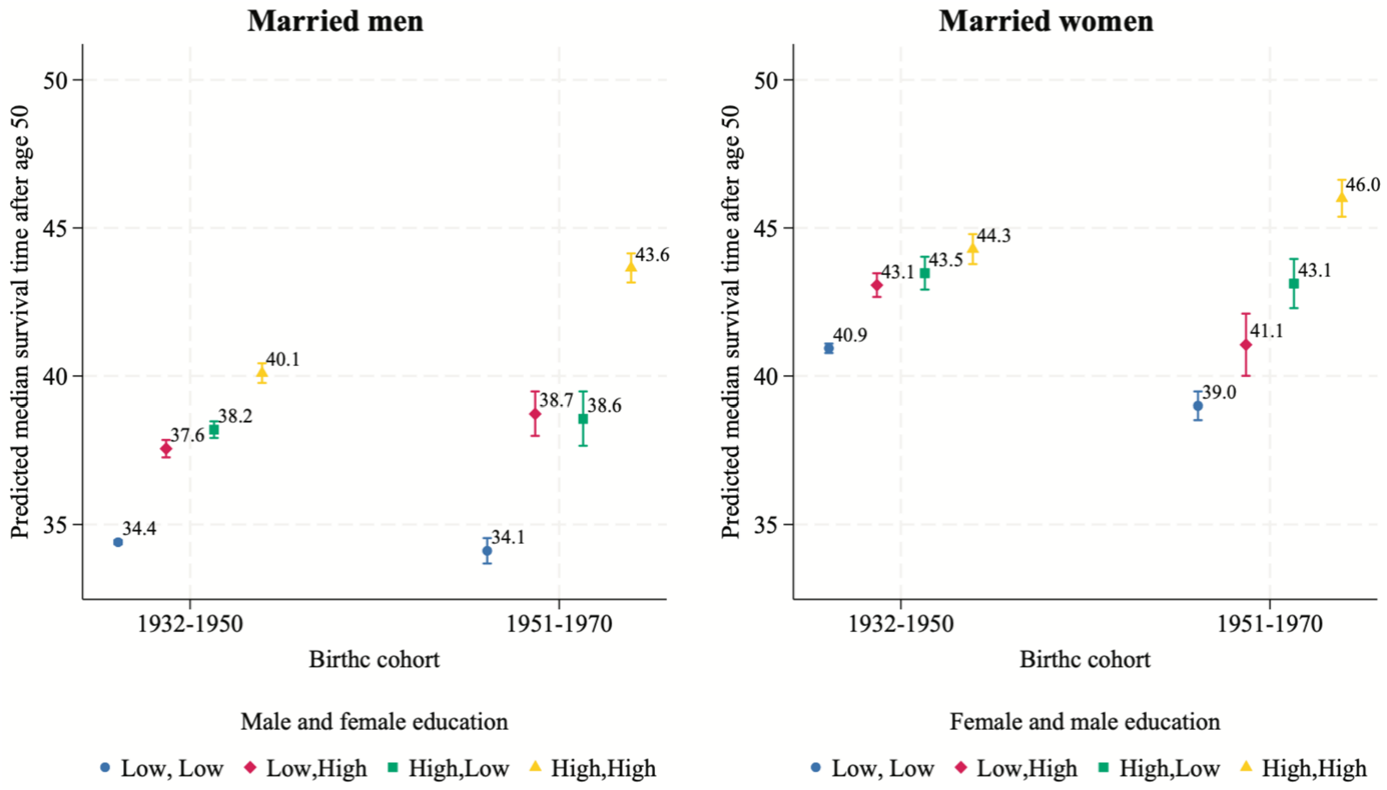
Predicted median time of survival for cohabiting individuals by educational pairing, gender and cohort. *Source*: Own computations from Statistics Finland register data (1987–2020). *Note*: (1) Confidence intervals are at the 95% level; (2) Numbers next to bars refer to median prediction. CIs available in Table 6

We now analyze the median survival time, namely, the time, starting at age 50, by when 50% of the individuals in the different combinations of couples are predicted to present the event of death. As shown in Figs. 4 and 5, although married women in heterogamous couples tend to have a significantly lower survival time than those in the highest educated pairings, the values are similar (43–44 years). These differences have, then, increased in the most recent cohort. This suggests that women’s mortality, in the first cohort, is influenced not only by their own education but also by their male partner’s education, and that this effect is more pronounced than it is for men. Across both birth cohorts, there is a significant increase in the predicted median survival time for married men and women in highly educated homogamous couples, alongside a decrease for one partner in homogamous low-educated couples. While the former increase is larger for men (3.5 vs 1.7 years), the latter decrease is far larger for women (0.3 vs 1.9 years).

Figure 5 shows the predicted median survival times for cohabiters. Across both birth cohorts, highly educated men and women in homogamous couples have a stable survival advantage, with cohabiting women in the earliest cohort being the only exception. Further, there is a significant increase in the mortality risk for women and men in low-educated couples (3.4 years less for men and more than 6.4 years less for women).

These results help explain the findings from Models 1 and 2 for cohabiting individuals and married women, which highlight the higher mortality among those born in 1951–1970 than those born in 1932–1950. This effect likely originates from mortality changes among people with the same educational level. As shown in Figs. 4 and 5, the health disadvantage of being low educated has largely increased in the later cohort, making the aggregate cohort effect appear negative. We addressed these cohort-level differences by including the three-way interaction in Model 3.

**Fig. 6:**
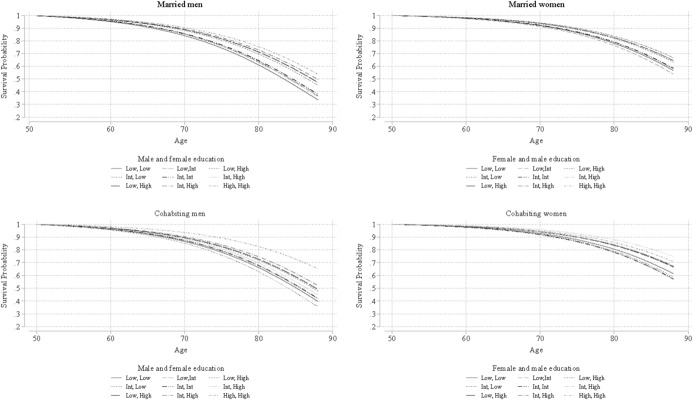
Survival curves for married and cohabiting individuals born in 1932–1950, by individual’s own education and partner’s education, including intermediate education (from Gompertz model). *Source*: Own computations from Statistics Finland register data (1987–2020)

**Fig. 7:**
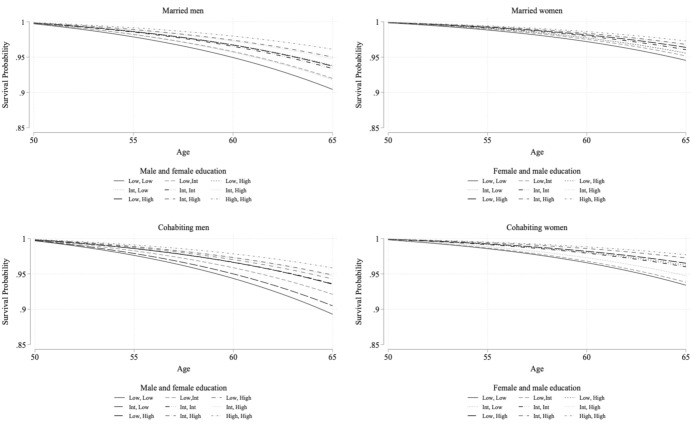
Survival curves for married and cohabiting individuals born in 1951–1970, by individual’s own education and partner’s education, including intermediate education (from Gompertz model). *Source*: Own computations from Statistics Finland register data (1987–2020)

As a further analysis, we also graphed our survival curves (Figs. 6 and 7 in the Appendix) showing intermediate education. The addition of such an education level did not change our conclusions. Indeed, couples with both intermediate partners or one intermediate partner and one low-educated partner had a lower survival probability than couples with one intermediate-educated partner and one highly educated partner. Cohabiting and married women presented the smallest differences across educational groups, thus aligning with previous results.

**Fig. 8:**
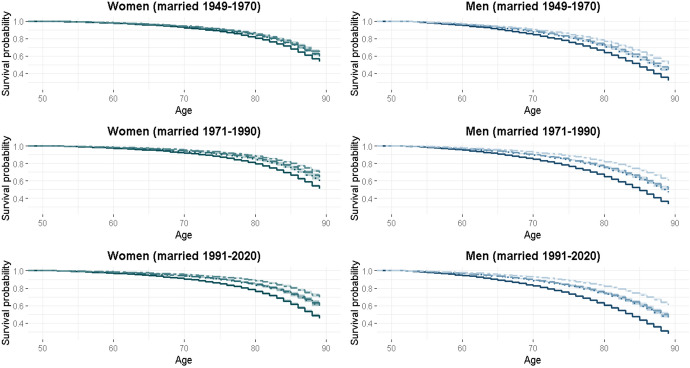
Survival curves for married men and women, by individual own education and partner’s education. *Source*: Own computations from Statistics Finland register data (1987–2020). *Note*: Confidence intervals were set at the 95% level

**Fig. 9:**
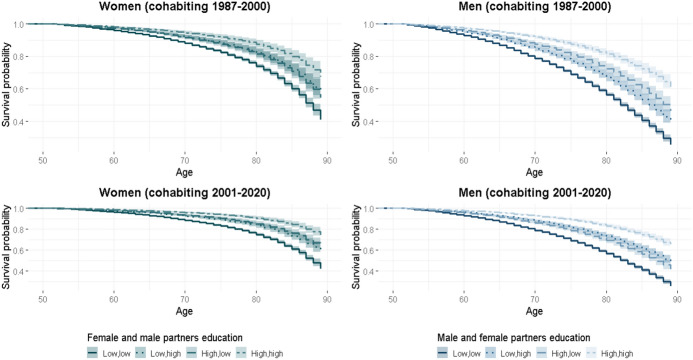
Survival curves for married men and women, by individual own education and partner’s education. *Source*: Own computations from Statistics Finland register data (1987–2020). *Note*: Confidence intervals were set at 95% level

Another robustness check consisted of running models stratified by marriage and cohabitation cohorts instead of birth cohorts to verify whether the year in which the coresidential union was established could lead to different conclusions. We established three marriage cohorts (1949–1970, 1971–1990, and 1991–2020) and two cohabitation cohorts (1987–2000 and 2001–2020) whose registration started later. The results are presented in the Appendix (Figs. 8 and 9). In all the cohorts, the mortality risk of married and cohabiting men as well as cohabiting women appears higher in the presence of educational resources, which is consistent with the resource multiplication mechanism. In contrast, the mortality rates of women who married between 1949 and 1970 were consistent with a resource substitution mechanism, and the following cohorts with resource multiplication.

**Fig. 10:**
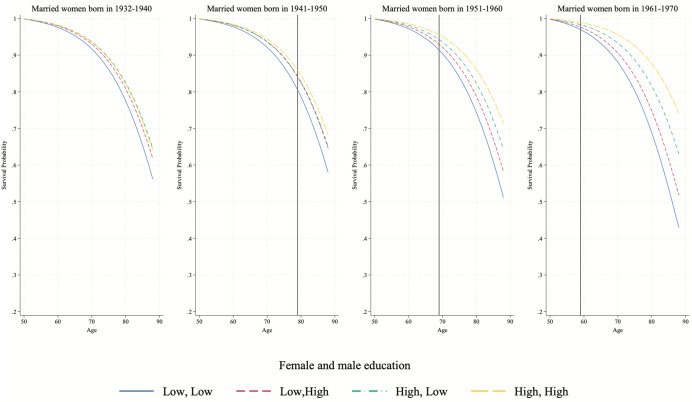
Survival curves for married women using a new cohort specification (10-year interval cohort) (from Gompertz survival model). *Source*: Statistics Finland (1987–2020). *Note*: the black line signals the beginning of the predictions, as the cohort has reached the maximum observational age

**Fig. 11:**
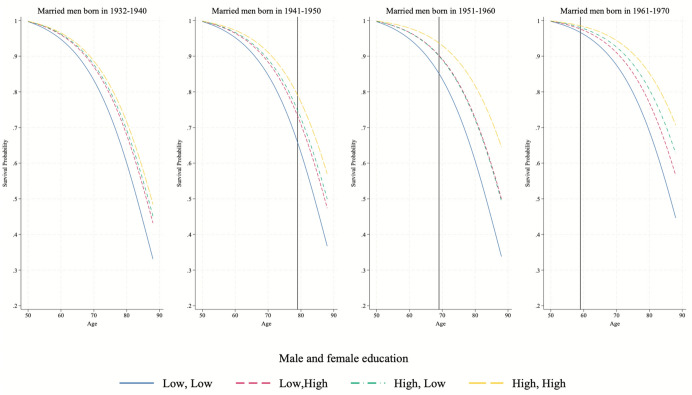
Survival curves for married men using a new cohort specification (10-year interval cohort) (from Gompertz survival model). *Source*: Statistics Finland (1987–2020). *Note*: the black line signals the beginning of the predictions, as the cohort has reached the maximum observational age

**Fig. 12:**
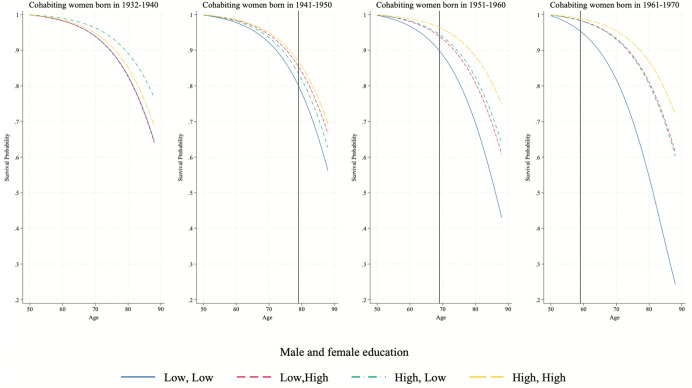
Survival curves for cohabiting women using a new cohort specification (10-year interval cohort) (from Gompertz survival model). *Source*: Statistics Finland (1987–2020). *Note*: the black line signals the beginning of the predictions, as the cohort has reached the maximum observational age

**Fig. 13:**
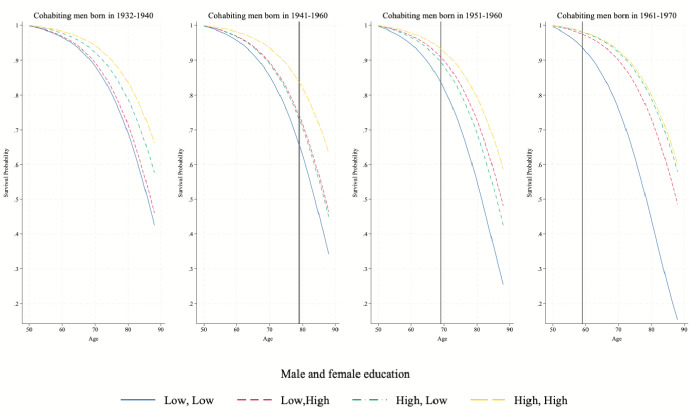
Survival curves for cohabiting men using a new cohort specification (10-year interval cohort) (from Gompertz survival model). *Source*: Statistics Finland (1987–2020). *Note*: the black line signals the beginning of the predictions, as the cohort has reached the maximum observational age

We also checked whether the results would majorly differ in case the cohorts were ten-year cohorts and not almost twenty years (Figs. 10, 11, 12 and 13). From the graphs, we do not see very large differences across adjacent cohorts, although there are some differences for the heterogeneous couples where the reference individual was born in 1951–1960 or 1961–1970, which are instead considered as a unique category in our paper. However, our interpretation of the results would not change. Across all the cohorts, we can see a progressive increase in the predicted mortality of the highly educated, compared to the other educational pairings. Conversely, the less educated tend to have a progressive disadvantage in terms of survival over time.

Another sensitivity analysis conducted on the birth cohorts consisted of using a continuous linear specification of birth cohort (results available upon request). The results of the continuous specification fit the trends of our dichotomous categorical variable. However, we observe that the mortality differences between women in highly educated homogamous couples and those in heterogamous couples are greater among women born in 1950 compared to those born in 1940. This suggests that the substitution mechanism observed among married women may already be diminishing for those born in the late 1940s.

## Discussion

Marked socioeconomic and demographic changes have occurred over recent decades. Average life expectancy has increased, although socioeconomic disparities in mortality have also widened (Marmot & Allen, 2014). Concurrently, women’s educational level and labor market participation have risen, resulting in greater educational assortative mating and changes in couples’ educational compositions (Schwartz & Mare, 2005). The postponement or abandonment of marriage, alongside the spread of cohabitation, has also underscored the need to examine this living arrangement more closely (Sassler & Lichter, 2020). Finland has experienced similar transformations, including the expansion of higher education, a significant rise in non-marital cohabitation, and growing socioeconomic inequalities in mortality.

Using Finnish register data, our study analyzes how the education of both partners in cohabiting and married couples is related to those individuals’ mortality risk across birth cohorts. The descriptive results highlight the extent to which the social context has changed over time. For example, the proportion of cohabitants is significantly higher in the latest considered cohort (1951–1970) than in the earliest (1932–1950). Another noticeable change pertains to the educational distribution among couples, with a significant expansion of education across both cohorts, from mostly low-educated pairs to more highly educated pairs. Furthermore, in line with Finland’s high level of gender equality, the educational changes of men and women are relatively similar regardless of their partner. Finally, the educational composition in cohabitation and marriage shows only minor differences.

Our analyses provide insights into whether the educational gradient among couples in distinct types of coresidential partnerships varies. Our first analysis aimed to verify H1, which proposed that a higher education for both the individual and their partner is associated with a higher individual survival probability. The hypothesis was supported, as we found a significant inverse association between both partners’ education and individual mortality risk. Both individuals and their partners having a higher education had a stronger association with reduced mortality than when only one partner in the couple had an intermediate education, suggesting that the higher both partners’ resources, the lower the individual’s mortality risk. Hence, among married individuals, the marginal effect of men’s intermediate and high education on their mortality is larger than it is for women’s. The contrary applies to cohabiting individuals. One possible explanation is that, being in a partnership, especially a marriage, is typically associated with higher earnings than being single. This premium is present for men but less for women, even in Finland (Geist & Reynolds, 2018; Ludwig & Brüderl, 2018).

Regarding the partner’s education, women’s intermediate and high education was more strongly associated with a reduction in men’s mortality than the reverse, within both marriage and cohabitation. This trend has previously been noted. For example, women are considered to more actively shape their male partner’s lifestyle than men, including influencing such choices as nutrition, health, and adherence to medical advice (Torssander & Erikson, 2009). Another explanation could be health selection, as highly educated women may prefer partners with healthier lifestyles (e.g., those that do not smoke or consume little or no alcohol). Simultaneously, men with unhealthy behaviors tend to avoid highly educated women with healthier lifestyles (Torssander & Erikson, 2009).

Our subsequent hypotheses sought to investigate whether married or cohabiting individuals differed in the associations observed across birth cohorts. In H2a, we proposed that in the earliest cohort born in 1932–1950, both women’s and men’s mortality risk would follow a “gendered” substitution mechanism, namely, decreasing when the man was highly educated and increasing when the man was less educated regardless of the woman’s educational level. This hypothesis was not supported.

For married men, mortality followed a resource multiplication mechanism: the more highly educated their partners, the higher their own survival probability. For married women, the mechanisms linking mortality and education were less straightforward. They were consistent with the resource multiplication mechanism, as being in a pairing in which both partners were highly educated was associated with women’s higher survival probability than being in a pairing in which only one partner was highly educated. Nevertheless, given the small gap between these three curves, a gender-neutral resource substitution mechanism is more likely to exist, meaning that both men’s and women’s education could compensate for the lack of education in one of the two partners.

H2b predicted that the survival probability of married individuals in the most recent birth cohort follows a resource multiplication pattern. We found that a multiplication effect was still present for men in this cohort. For women, the differences in mortality risk between those in highly educated couples and those in couples with at least one low-educated partner increased compared with in the previous cohort. However, these differences were more moderate than those for men. This sex difference confirms the lower reliance of women on their male partner’s education, in line with previous health studies arguing that men rely more heavily on their female partners’ education than the reverse (Skalická & Kunst, 2008; Torssander & Erikson, 2009). Overall, these results underscore the longstanding significance of women’s education in married Finnish households regardless of the identified mechanism (Husu, 2007; Martikainen, 1995).

H3 predicted a multiplication mechanism regulating mortality for cohabiters regardless of their sex and birth cohort. Cohabitation, which is generally viewed as being based on more egalitarian gender norms than marriage, serves as a setting for more homogamous couples (Laufer & Gemici, 2011; Schoen & Weinick, 1993). However, some studies have challenged this perspective, emphasizing that marriage and cohabitation are likely to exhibit similar mechanisms, as cohabitation is often considered an “informal marriage” (Blackwell & Lichter, 2000; Verbakel & Kalmijn, 2014). Indeed, an increasing number of individuals started to adopt cohabitation as an alternative to marriage in the second half of the twentieth century across Western countries, with Nordic countries being the forerunners (Sobotka & Berghammer, 2021).

In the earliest cohort, we found that men’s survival was related to their partners’ education through a resource multiplication mechanism, while women’s survival was related through a substitution mechanism. This latter result contradicts our expectations but aligns with what was observed among married individuals born in the earliest cohort. The results for the more recent cohort confirmed the presence of a multiplication effect for both men and women, consistent with that witnessed for the most recent cohort of married women. As the distribution of socioeconomic resources in mortality is similar for married and cohabiting individuals, the results seem to support the “informal marriage” argument. Further, since cohabitation has increasingly become a premarital phase, married individuals may have been former cohabiters, thus presenting similar characteristics to cohabiting individuals. However, although the mechanisms underlying socioeconomic resources are similar for marriage and cohabitation, we cannot establish the full equivalence between these two coresidential partnership types.

Looking at the long-term predictions of the median survival time, differences in SES tend to increase over time differently by the type of union. Among married individuals, the median survival time has significantly risen for those in couples in which both partners are highly educated, especially for men. Women in low-educated couples have witnessed a similar decline in survival. Likewise, among cohabiters, the predicted median survival time for couples in which both partners are low educated has declined. This result likely highlights two aspects. First, educational disparities in mortality have widened in Finland, in line with the trends in several other Western countries (Long et al., 2023; Montez et al., 2019). Second, these results are in line with demographic studies highlighting that marriage has become progressively more socioeconomically differentiated than in the past: the most educated and healthiest individuals tend to marry, whereas cohabitation at older ages could signal negative selection (Dykstra, 2021; Moustgaard & Martikainen, 2009).

Our study provides new evidence on couple-level inequalities in mortality. However, it is not without limitations. First, we present a descriptive analysis of the association between a partner’s educational level and individual mortality. Nonetheless, these results may be influenced by unobserved confounding factors that affect both education and mortality, such as the family backgrounds of both partners. Second, the samples only included individuals who were in partnerships during the study period. The process of selection into a partnership may have changed, leading to us studying only a selected group of the population (Jalovaara, 2012). Further, the conclusions about cohabitation are limited by the fact that cohabitation can only be inferred from 1987. Moreover, the role of partner resources on mortality was evaluated in a younger population than the married population. Another issue is that the relationships among cohabitation, SES, and mortality can change at young and older ages (Lindmarker et al., 2025), as the meaning of cohabitation changes across age groups. While cohabitation without marrying is more common at young ages, it may signal socioeconomic disadvantage and be related to higher mortality in late adulthood. Conversely, cohabitation after separating or becoming widowed is more common among older people and characterized by a similar level of mortality as the married. Although this result has only been verified in Sweden, a similar effect could be expected in Finland. However, while we acknowledge the different meanings of cohabitation over the life course, it is difficult to disaggregate the analysis on cohabiters further.

From a policy perspective, health spillover effects within families are a major area for future research. Recognizing education as a beneficial resource for multiple family members underscores its broader societal benefits. Additionally, by offering insights into how socioeconomic resources influence health at the couple level, it may help identify new health vulnerabilities not only at the individual level but also at the family level. Recognizing these factors allows the development of targeted interventions that can mitigate some of the associated risks. Men with low educational levels, partnered with women with low educational levels, represent an increasingly disadvantaged group. Public health initiatives should focus on compensatory strategies to minimize the health differences linked to educational disadvantages in couples.

Future research should aim to better understand the mechanisms by which these inequalities emerge and evolve. For example, there should be a clearer understanding of whether psychosocial pathways, income, and wealth contribute to the observed results. While income and wealth are important factors to consider, they are possible mediators of the educational effects examined in this study. Investigating their role would require a distinct theoretical framework and analytical approach, which could be a valuable direction for future research. Furthermore, so far, our study has only focused on a selected population, namely couples. Partnership selection is a crucial process that must first be considered. Future studies should explore the conditions under which a partnership health advantage exists. Moreover, a better understanding of the consequences of changing assortative mating patterns is essential for future studies on population health.

## Data Availability

The data supporting the findings of this study are available from Statistics Finland. Restrictions apply to the availability of these data, which were used under permission. TK/2181/07.03.00/2024.

## Appendix

See Tables 5, 6 and Figs. 6, 7, 8, 9, 10, 11, 12 and 13.

**Table 5:** Tests of the proportional hazard assumption of Cox regressions for men and women, by coresidential union

	Cohabiting		Married	
	Test statistics	*P*-value	Test statistics	*P*-value
Women	${\chi }^{2}\left(17\right)=24.9$	0.10	${\chi }^{2}\left(17\right)=26.13$	0.07
Men	${\chi }^{2}\left(17\right)=27.95$	0.05	${\chi }^{2}\left(17\right)=154.3$	0.00

*Source*: Own computations from Statistics Finland data (1987–2020)

We computed the tests on Model 3 in both Tables 3 and 4

**Table 6:** Estimates of median survival times, including confidence intervals

Estimated median	Lower CI	Upper CI	Own education	Partner's education	Birth cohort
*Cohabiting men*					
36.11	35.80	36.42352	Low	Low	1932–1950
32.73	31.92	33.54607	Low	Low	1951–1970
38.39	37.46	39.30762	Low	High	1932–1950
39.29	37.63	40.95021	Low	High	1951–1970
38.74	37.54	39.93082	High	Low	1932–1950
38.02	36.14	39.89153	High	Low	1951–1970
43.90	42.12	45.68039	High	High	1932–1950
42.62	41.13	44.09645	High	High	1951–1970
*Cohabiting women*					
42.31	41.78	42.83309	Low	Low	1932–1950
35.94	35.01	36.8632	Low	Low	1951–1970
44.00	42.66	45.3498	Low	High	1932–1950
41.02	38.72	43.31568	Low	High	1951–1970
43.87	42.14	45.59844	High	Low	1932–1950
41.62	39.86	43.37622	High	Low	1951–1970
45.20	42.80	47.60867	High	High	1932–1950
45.35	43.49	47.21238	High	High	1951–1970
*Married men*					
34.40	34.33	34.47158	Low	Low	1932–1950
34.11	33.68	34.53998	Low	Low	1951–1970
37.56	37.27	37.85503	Low	High	1932–1950
38.73	37.99	39.47545	Low	High	1951–1970
38.20	37.92	38.48509	High	Low	1932–1950
38.57	37.66	39.47629	High	Low	1951–1970
40.09	39.76	40.42658	High	High	1932–1950
43.65	43.15	44.13939	High	High	1951–1970
*Married women*					
40.93	40.77	41.09312	Low	Low	1932–1950
39.00	38.52	39.47586	Low	Low	1951–1970
43.07	42.67	43.46667	Low	High	1932–1950
41.05	40.00	42.10372	Low	High	1951–1970
43.47	42.92	44.02469	High	Low	1932–1950
43.12	42.29	43.95008	High	Low	1951–1970
44.28	43.78	44.78853	High	High	1932–1950
46.00	45.38	46.62406	High	High	1951–1970
